# Multi-Frequency Microwave Sensing System with Frequency Selection Method for Pulverized Coal Concentration †

**DOI:** 10.3390/s24227245

**Published:** 2024-11-13

**Authors:** Haoyu Tian, Feng Gao, Yuwei Meng, Xiaoyan Jia, Rongdong Yu, Zhan Wang, Zicheng Liu

**Affiliations:** 1 Zhejiang Energy Digital Technology Co., Ltd., Hangzhou 310012, China; tianhaoyu@zjenergy.com.cn (H.T.); gaofeng6@zjenergy.com.cn (F.G.); mengyw@zjenergy.com.cn (Y.M.); jiaxiaoyan@zjenergy.com.cn (X.J.); yurongdong@zjenergy.com.cn (R.Y.); 2 School of Electronics and Information, Northwestern Polytechnical University, Xi’an 710072, China

**Keywords:** pulverized coal concentration, frequency selection, microwave sensor, switch matrix, principal component analysis (PCA), orthogonal matching pursuit (OMP)

## Abstract

The accurate measurement of pulverized coal concentration (PCC) is crucial for optimizing the production efficiency and safety of coal-fired power plants. Traditional microwave attenuation methods typically rely on a single frequency for analysis while neglecting valuable information in the frequency domain, making them susceptible to the varying sensitivity of the signal at different frequencies. To address this issue, we proposed an innovative frequency selection method based on principal component analysis (PCA) and orthogonal matching pursuit (OMP) algorithms and implemented a multi-frequency microwave sensing system for PCC measurement. This method transcended the constraints of single-frequency analysis by employing a developed hardware system to control multiple working frequencies and signal paths. It measured insertion loss data across the sensor cross-section at various frequencies and utilized PCA to reduce the dimensionality of high-dimensional full-path insertion loss data. Subsequently, the OMP algorithm was applied to select the optimal frequency signal combination based on the contribution rates of the eigenvectors, enhancing the measurement accuracy through multi-dimensional fusion. The experimental results demonstrated that the multi-frequency microwave sensing system effectively extracted features from the high-dimensional PCC samples and selected the optimal frequency combination. Filed experiments conducted on five coal mills showed that, within a common PCC range of 0–0.5 kg/kg, the system achieved a minimum mean absolute error (MAE) of 1.41% and a correlation coefficient of 0.85. These results indicate that the system could quantitatively predict PCC and promptly detect PCC fluctuations, highlighting its immediacy and reliability.

## 1. Introduction

In coal-fired power plants, the combustion conditions within boilers are intricately linked to the conveying conditions of pulverized coal in each burner. Factors such as the velocity and concentration of pulverized coal, as well as the uniformity of the air–coal mixture in individual burners, directly affect combustion stability and boiler efficiency [1]. Issues such as flame skewing and unstable ignition may lead to significant economic losses and compromise plant safety. Achieving optimal boiler combustion requires meticulous real-time control of the pulverized coal parameters, which includes the precise measurement of the airflow and the pulverized coal flow.

In power plants, pulverized coal flow is classified as dilute-phase gas–solid two-phase flow. Influenced by pipeline geometric, air temperature and humidity, and the physical properties of coal particles, the flow exhibits diverse patterns and random distributions, making the process highly complex [2]. Consequently, measuring the parameters of pulverized coal flow presents substantial challenges. Over the years, researchers have made various attempts to address these challenges. Baucum et al. [3] employed Coriolis flow meters in the 1970s to measure dense-phase coal flows. Gu et al. [4] utilized a dual-frequency ultrasound for the online detection of coal particle volume fractions and sizes, while Wang et al. [5] applied electrical capacitance tomography (ECT) to mitigate the impact of gas–solid flow patterns and measure pulverized coal concentration (PCC). Cai et al. [6] analyzed particle size, average particle number density, and concentration, using light transmission fluctuation. With the advancement of computing techniques, researchers have leveraged advanced algorithms and models to enhance measurement processes [7]. For instance, Wang et al. [8,9] integrated adaptive wavelet and adaptive fuzzy neural networks to enhance electrostatic and capacitive sensors for measuring coal/biomass/air three-phase flows. Jin et al. [10] introduced a digital holographic particle analyzer (DHPA) for the three-dimensional measurement of fuel particles using pulsed digital inline holography.

Microwave technology, successfully applied in various industrial contexts, has also been explored for the measurement of pulverized coal. Methods include transmission methods, resonant cavity methods, microwave tomography (MWT) methods, and Doppler methods. Transmission methods [11,12,13] estimate solid particle concentration based on the propagation loss of microwave signals after passing through the space of pulverized coal. For instance, Abou-Khousa et al. [14] detected and quantified solid contaminants (such as black powder) in gas flows by measuring the power and phase shifts in transmitted and reflected signals. Resonant cavity methods [15] utilize phase shifts induced by the changes in effective permittivity at the resonance frequencies to determine the solid concentrations. Andreas et al. [16] proposed a material density sensor based on two novel microstrip–patch (MSP) couplers for detecting changes in the density of polyethylene (PE) powder in the air. Wang et al. [17] introduced the concept of normalized resonant frequency as a means to measure the solid concentration in gas–solid two-phase flows. MWT reconstructs spatial distributions of permittivity using multiple transmitting and receiving antennas, initially applied in medical research before being adapted for the industrial imaging of oil–gas two-phase flows [18,19]. Doppler methods [20,21] typically detect particle velocities by measuring frequency shifts in reflected microwave signals from gas–solid two-phase flows.

Microwave techniques are non-invasive and can enhance the reliability, stability, and representativeness of PCC measurement. Common microwave measurement methods typically employ a single frequency [15,19,20,21,22]. However, variations in signal sensitivity at different frequencies may affect measurement results. Consequently, microwave characteristics, such as attenuation and phase shift, yield different results at various frequencies, suggesting that multi-frequency measurements can provide more comprehensive information [23], although excessive frequencies may introduce redundancy. Therefore, effective methods for frequency selection are essential. Table 1 summarizes similar efforts made by other researchers.

In this paper, a frequency selection method for the PCC sensing system is presented. By applying dual-dimensionality reduction through principal component analysis (PCA) and orthogonal matching pursuit (OMP), this data-driven method analyzes the complex features of extensive frequency signals and evaluates the contribution of each frequency signal to develop a multi-frequency microwave sensing system for optimizing PCC measurement at power plants.

## 2. PCC-Sensing Mechanism

**Figure 1:**
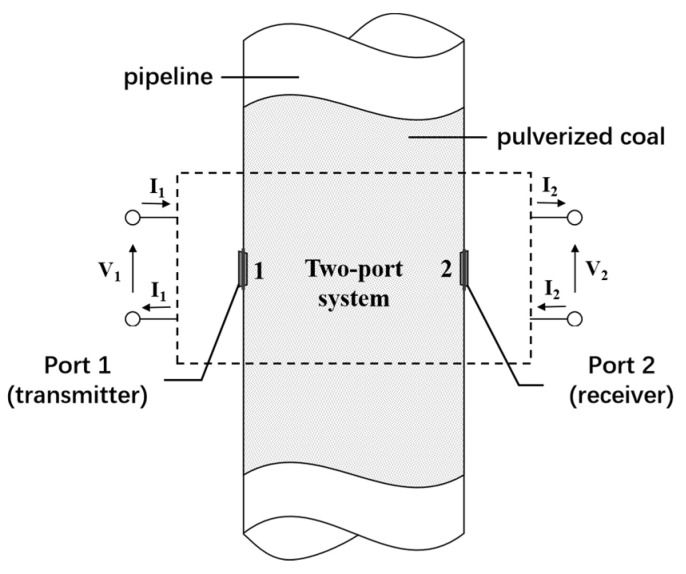
Two-port waveguide system for coal pipeline.

As illustrated in Figure 1, the coal pipeline for pulverized coal flow can be regarded as a two-port guided wave system. By placing a transmitter (Port 1) on the pipeline, the transmitted wave passes through pulverized coal particles and is received by the receiver at Port 2. Due to the lossy property of PCC, the transmitted wave is attenuated, and the attenuation is proportional to the concentrations of pulverized coal. Such a fact allows the determination of PCC based on wave attenuation. The attenuation is quantified by the ratio of the receiving power at Port 2 to the emitting power at Port 1, i.e., the insertion loss S_21_.

We denoted the power emitted by the transmitter as $P\left(0\right)$. The received power at distance *z* from the source could be quantified by

(1) $$P\left(z\right)=P\left(0\right)e^{-2\alpha z}$$

where *α* is the attenuation constant, which depends on the frequency and the electromagnetic parameter (e.g., conductivity) of the medium. The expression for *α* is [25]:

(2) $$\alpha =\frac{\omega }{c}\sqrt{\frac{\varepsilon_{r}}{2}\left(\sqrt{1+{\left(\frac{\sigma }{\omega \varepsilon_{r}\varepsilon_{0}}\right)}^{2}}-1\right)}$$

where ω is the angular frequency, c is the wave speed, $\varepsilon_{r}$ is the relative permittivity of the medium, σ is the conductivity of the medium, and $\varepsilon_{0}$ is the permittivity of free space.

The pneumatic conveying process for pulverized coal is a typical gas–solid two-phase flow, which is inherently complex, making the study of the electromagnetic behavior of such multiphase systems challenging. We introduced the macroscopic dielectric constant to analyze the pulverized coal conveying process. The macroscopic dielectric constant incorporated the geometric parameters of the mixture, such as the dielectric constant and volume ratio of the material components. The heterogeneous mixture could be electromagnetically equivalent to a homogeneous medium with the dielectric constant for two-phase mixtures [26]:

(3) $$\varepsilon_{m}=\left(1-\Delta V_{s}\right)\varepsilon_{g}+\Delta V_{s}\varepsilon_{s}$$

where $\Delta V_{s}$ is the volume ratio of the solid phase in the mixture, $\varepsilon_{g}$ is the dielectric constant of the gas phase, and $\varepsilon_{s}$ is the solid phase.

Deriving the equation for the volume ratio of the solid phase, $\Delta V_{s}$ yields the following formula:

(4) $${\varepsilon_{m}}^{\prime }=\varepsilon_{s}-\varepsilon_{g}$$

Since the dielectric constant of solids is generally higher than that of gasses, the dielectric constant of a gas–solid mixture positively correlates with the volume ratio of its solid phase. As the volume ratio of solid phase increases, the macroscopic dielectric constant of the gas–solid mixture increases, resulting in the stronger attenuation effect of the microwave signal as it traverses the solid phase. Moreover, combining Equation (2), the attenuation constant α can be expressed as a function $f\left(x\right)$ related to $\varepsilon_{m}$. Due to the correlation between $\varepsilon_{m}$ and $\Delta V_{s}$, the function g(x) can be employed to represent the mapping relationship between α and $\Delta V_{s}$, as shown in the following equation:

(5) $$\alpha =K\cdot f\left(\varepsilon_{m}\right)=K\cdot g(\Delta V_{s})$$

where *K* is a characteristic parameter of the PCC sensor, determined by the sensor’s dimensions, structure, and other attributes.

Given that the insertion loss S_21_ is directly linked to the attenuation constant, it indicates an indirect relationship between the insertion loss S_21_ of the two-port system and the volume ratio of the solid phase. Therefore, by extracting the characteristics of the insertion loss S_21_, we can analyze sensor data to reveal the abstract relationship. Consequently, this enables the inference of solid phase concentration at the measured cross-section based on the measured S_21_ data of the two-port system.

## 3. Materials and Methods

**Figure 2:**
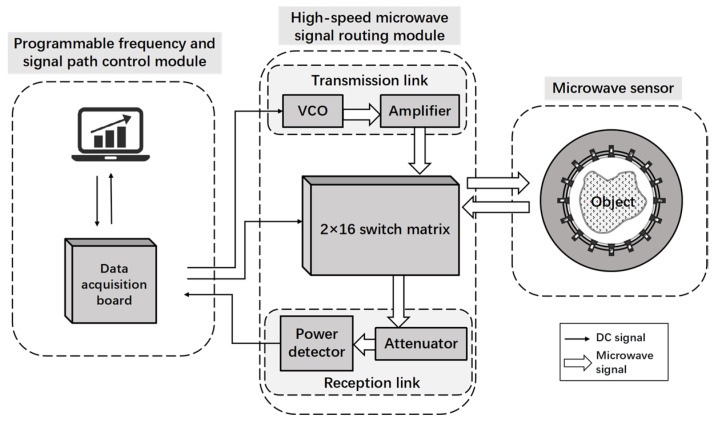
Overall framework of the multi-frequency microwave sensing system.

The overall framework of the proposed PCC measurement system is illustrated in Figure 2. The system is composed of three main components: a microwave sensor, a high-speed microwave signal routing module, and a programmable frequency and signal path control module. The microwave sensor is made of 16 electrodes which can emit or collect the electromagnetic waves, while the emitting and collecting function can be altered by a switch matrix. The high-speed microwave signal routing module is responsible for amplification, noise-reduction, filtering, and analog-to-digital conversion. The programmable frequency and signal path control module communicates bidirectionally with the high-speed microwave signal routing module. It has the function of setting working frequency and signal path.

### 3.1. Design of Microwave Sensor

**Figure 3:**
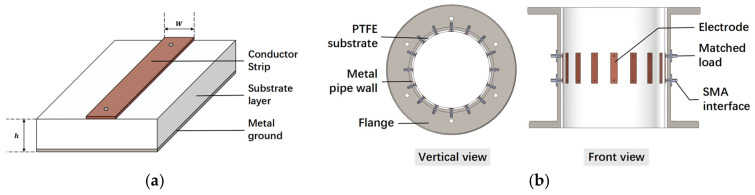
Schematic structure: (**a**) typical microstrip line; (**b**) microwave sensor.

The metal electrodes constitute the core unit of the sensor, primarily responsible for transmitting and receiving microwave signals to establish a signal path. A polytetrafluoroethylene (PTFE) substrate layer, positioned beneath the electrodes, securely anchors them in place. Each electrode is soldered to two SMA connectors, which connect to the SMA cable and matching load, respectively. In microwave signal transmission lines, impedance matching involves adjusting the load impedance to match the internal impedance of the excitation source, thereby maximizing the device’s output power. The dimensions of the electrodes and the PTFE substrate layer are tailored to meet the 50-ohm characteristic impedance requirements, ensuring efficient microwave signal transmission. The microstrip line depicted in Figure 3a is a commonly used transmission line, with its characteristic impedance described by the following formula [27]:

(6) $$\varepsilon_{re}=\frac{\varepsilon_{r}+1}{2}+\frac{\varepsilon_{r}-1}{2}{\left(1+12\frac{h}{W}\right)}^{-\frac{1}{2}}$$

(7) $$Z_{0}=\frac{120\pi }{\sqrt{\varepsilon_{re}}\left[\frac{W}{h}+1.393+0.667\ln\left(\frac{W}{h}+1.444\right)\right]}$$

where $\varepsilon_{re}$ is the equivalent dielectric constant of the dielectric substrate, $\varepsilon_{r}$ and h are the dielectric constant and height of the dielectric substrate, respectively, *W* is the width of the conductor band, and $Z_{0}$ is the characteristic impedance of the microstrip transmission line.

The sensor proposed in this paper, as illustrated in Figure 3b, is primarily designed for tomography techniques [23,28]. Its ring shape allows it to directly replace a section of a coal pipeline in practical applications. The sensor consists of metal electrodes, a PTFE substrate layer, SMA connectors, a flange, and a metal outer wall. The metal outer wall, constructed from carbon steel, provides mechanical support and protection while also functioning as a grounding layer for microwave signals, thereby mitigating the effects of external electromagnetic interference. Furthermore, the sensor meets the necessary requirements for wear resistance, heat resistance, and non-intrusiveness.

### 3.2. High-Speed Microwave Signal Routing Module

The high-speed microwave signal routing module comprises a 2-by-16 switch matrix and a microwave signal conditioning module. The 2-by-16 switch matrix interfaces the RF cable with the 16 electrodes of the sensor via SMA connectors, and then adjusts the switching states by controlling the transmission and reception links in the microwave signal conditioning module.

**Figure 4:**
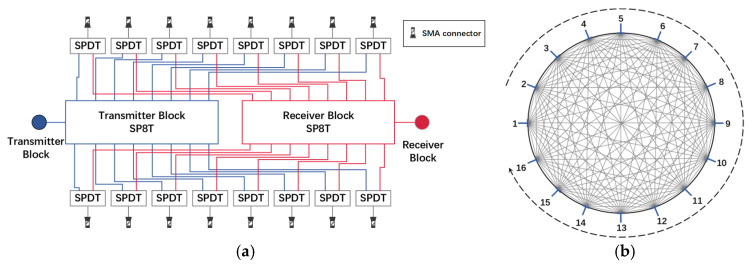
(**a**) Schematic diagram of the 2-by-16 switch matrix; (**b**) electrode pair combinations at 16 electrodes.

The 2-by-16 switch matrix consists of two 1-by-2 switching circuits and two 2-by-8 switching circuits [29,30]. The 1-by-2 switching circuits connect to either the transmission link or the reception link, while the 2-by-8 switching circuits select specific signal paths and determine the direction of signal flow. The signals are routed through various paths between the electrodes, as illustrated in Figure 4a.

The SPDT RF switch and the 3-to-8-line decoder demultiplexer used in the circuit are detailed in Table 2. These components exhibit extremely fast switching times of 6 ns and 12 ns, respectively, enabling the effective measurement of the rapid flow of pulverized coal.

During system operation, one pair of electrodes is designated as the transmitter and receiver, forming a signal path. By switching the signal propagation path, a total of $C_{16}^{2}=120$ electrode pairs can be generated. Considering the hardware asymmetry, it is necessary to alternate the transmitter and receiver, resulting in a total of $A_{16}^{2}=240$ distinct electrode pairs and corresponding signal paths, as illustrated in Figure 4b. The abundance of signal paths allows the measurement area to transition from a linear to a surface format, thereby covering a larger area. For the turbulence phenomena that may occur in pulverized coal flow [31], the high speed and full-path measurement of this approach effectively mitigates the uneven distribution of pulverized coal and addresses the low sensitivity of the central electric field, leading to more accurate measurements.

The conditioning of microwave signals primarily depends on the transmission and reception links. The transmission link regulates the output frequency and the power of the microwave signal through a voltage-controlled oscillator (VCO) and an amplifier. Due to the varying sensitivity of the signals across different frequency bands, changes in the working environment may lead to fluctuations in measurement performance. Therefore, prior to deployment, it is essential to identify the frequency that best suits the current operating conditions, ensuring effective noise suppression and optimal measurement results. The reception link consists of an attenuator and a power detector, which are primarily used to measure the amplitude of the received signal. The power detector employed is the AD8362 chip (Analog Devices, Norwood, MA, USA), capable of detecting microwave signals with a minimum signal strength of −55 dBm.

### 3.3. Programmable Frequency and Signal Path Control Module

The module integrates a host computer with a Data Acquisition card (DAQ). To mitigate dust pollution in industrial environments, the host computer employs a specialized industrial computer designed to withstand a wide range of operating temperatures, humidity, vibrations, and dust, ensuring long-term reliable performance. Additionally, software has been developed to issue hardware control commands, managing the system’s working frequency and signal path while receiving and processing sensor data. It facilitates visual interaction with the user. The DAQ executes commands from the host computer and provides bidirectional communication between the hardware circuits and the host computer. The PCIE-6361 (National Instruments, Austin, TX, USA) is employed as the DAQ, offering exceptionally high sampling and data transfer rates to meet the system’s high-speed read and write requirements.

The high-dimensional PCC dataset contains full-path data across multiple frequencies, which makes direct computation highly complex and challenging to interpret intuitively. To address this, we implemented a dual-dimensionality reduction approach using PCA and OMP. PCA is used to reduce the dimensionality of the full-path data, while OMP selects the optimal frequency.

PCA aims to identify the new variables that capture the primary features of the dataset, effectively compressing the original data matrix and retaining the minimum number of dimensions that represent the most significant characteristics. During PCA, the process involves calculating the covariance matrix and deriving its eigenvalues and corresponding eigenvectors. Each eigenvector corresponds to a principal component, representing the projection of the original data onto the eigenspace. The *i*-th principal component ${PC}_{i}$ can be expressed by the following formula:

(8) $${PC}_{i}=\sum_{j}a_{ij}x_{j}$$

where $a_{ij}$ is the weight of the *i*-th principal component on the *j*-th original dimension, and $x_{j}$ is the *j*-th dimension of the original data.

The information content of the *i*-th principal component, i.e., the cumulative explained variance, is calculated as the ratio of its eigenvalue to the sum of all the eigenvalues. The number of principal components, k, can be determined based on the desired level of cumulative explained variance.

After applying PCA, a reduced number of principal components is retained for each frequency. However, due to the large number of frequencies, directly using iterative methods to select the optimal frequency combination may still risk computational explosion. OMP is a sparse signal reconstruction algorithm based on a greedy strategy that constructs a sparse representation of a signal by iteratively selecting the dictionary atoms most relevant to the residuals. In each iteration, OMP updates the residuals and coefficients through orthogonal projections, ensuring orthogonality and stability among the selected atoms. Therefore, OMP can effectively mitigate the risk of computational explosion. For the PCC dataset, the objective is to determine a combination S of frequency signals from a matrix $A\in R^{t\times v}$ of multiple sets of eigenvectors such that the predicted PCC $y\in R^{t}$ closely matches the actual PCC of the coal pipeline. During computation, the eigenvector with the largest inner product with a residual vector is continuously selected and added to the frequency combination $S\leftarrow S\cup \left\{i\right\}$. Subsequently, the optimal eigenvector $x_{S}={\left(A_{s}^{T}A_{S}\right)}^{-1}A_{S}^{T}y$ is computed using the least squares method based on the eigenvectors in the current frequency combination S to make $A_{S}x_{S}$ approximate the actual PCC as closely as possible, where $A_{S}$ is a sub-matrix consisting of the eigenvectors in the combination S.

## 4. Experiments and Results

### 4.1. Prototype Experiments

**Figure 5:**
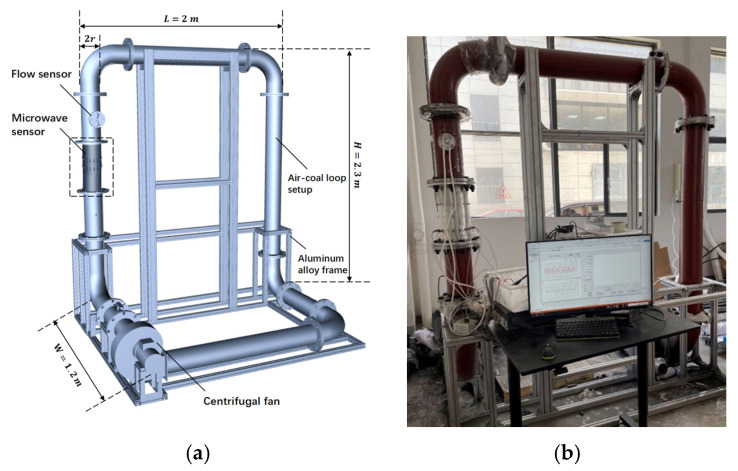
The air–coal loop setup: (**a**) simulation model; (**b**) actual installation.

To simulate the working conditions of pulverized coal flow, we constructed an air–coal loop setup [32]. This setup, depicted in Figure 5, was designed as a prototype system to investigate the interaction between microwave signals and pulverized coal. The setup was supported by an aluminum alloy frame, with the microwave sensor mounted at the center of the left steel pipe via a flange. The air–coal loop setup facilitated the circulation of pulverized coal using an industrial fan. A centrifugal fan with a power rating of 750 W and a nominal flow rate of 1810 m^3^/h served as the power source for air circulation. Under this configuration, the internal flow velocity of the setup could reach up to 28.4 m/s, meeting the actual flow velocity requirements of 20–30 m/s.

**Figure 6:**
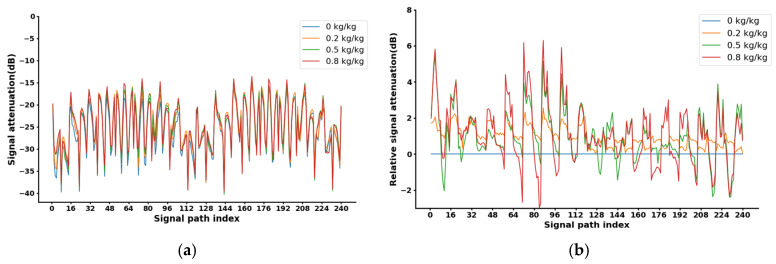
Measurement of different concentrations: (**a**) raw signal attenuation; (**b**) relative signal attenuation.

The pulverized coal concentration in power plants typically ranges from 0.2 to 0.8 kg/kg. Accordingly, we set the coal concentration in the air–coal loop setup to 0.2, 0.5, and 0.8 kg/kg for the measurements. During each measurement, each electrode was sequentially paired with the remaining electrodes, allowing for the collection of 240 S21 data points across the entire cross-section, as shown in Figure 6a. Subtracting the 0 kg/kg measurement value from each concentration’s measurement yielded elative signal attenuation, as shown in Figure 6b. It is evident that a distinct stratification occurred at each concentration, with higher concentrations displaying greater deviation from the zero baseline. This supports our hypothesis of a mapping relationship between coal concentration and microwave attenuation.

During the prototype experiments, we employed the L1 distance metric to preliminarily establish the mapping relationship between coal concentration and signal attenuation. The L1 distance, also known as the Manhattan distance, is a method used to measure the distance between two points in a space. In our case, we treated multiple signal paths as separate dimensions, which enabled the calculation of the L1 distance relative to 0 kg/kg and the determination of coal concentration based on this value. The formula for the L1 distance metric is expressed as follows:

(9) $$f\left(X\right)=D\left(X,Y\right)=\sum_{i=1}^{n}\left|x_{i}-y_{i}\right|$$

where $x_{i}$ is the S_21_ data under various concentration conditions, $y_{i}$ is the S_21_ data under the 0 kg/kg condition, and i is the signal path index.

**Figure 7:**
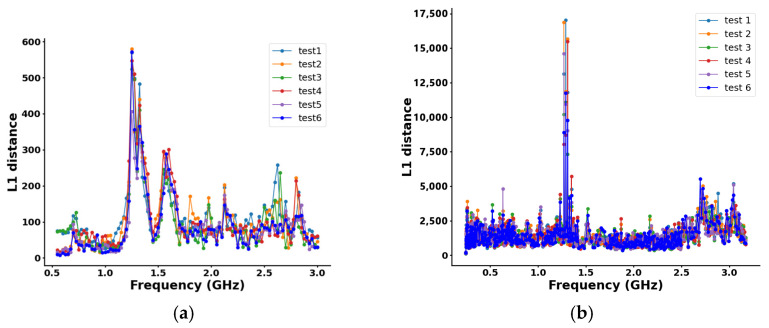
Distribution of L1 distance values at each frequency: (**a**) prototype experiments; (**b**) field experiments.

We used five VCOs to sweep the microwave signal from 0.5 to 3 GHz in steps of 0.025 GHz. For the full-path S21 data corresponding to a PCC of 0.8 kg/kg, the L1 distance metric calculation results were presented in Figure 7a. After analysis, the correlation coefficients for each experimental group, calculated based on test 1, are shown in Table 3. It can be observed that the correlation coefficient for each group was very close to 1, indicating a high degree of similarity and suggesting that these experiments are reproducible. It can be concluded that, under this specific condition, the microwave signal at approximately 1.25 GHz exhibited the peak L1 distance metric, indicating that microwave signals near this frequency band experience the strongest attenuation after passing through the pulverized coal flow. Therefore, with consistent hardware and measurement environments, it is evident that selecting the appropriate working frequency was crucial for obtaining accurate measurement results.

**Figure 8:**
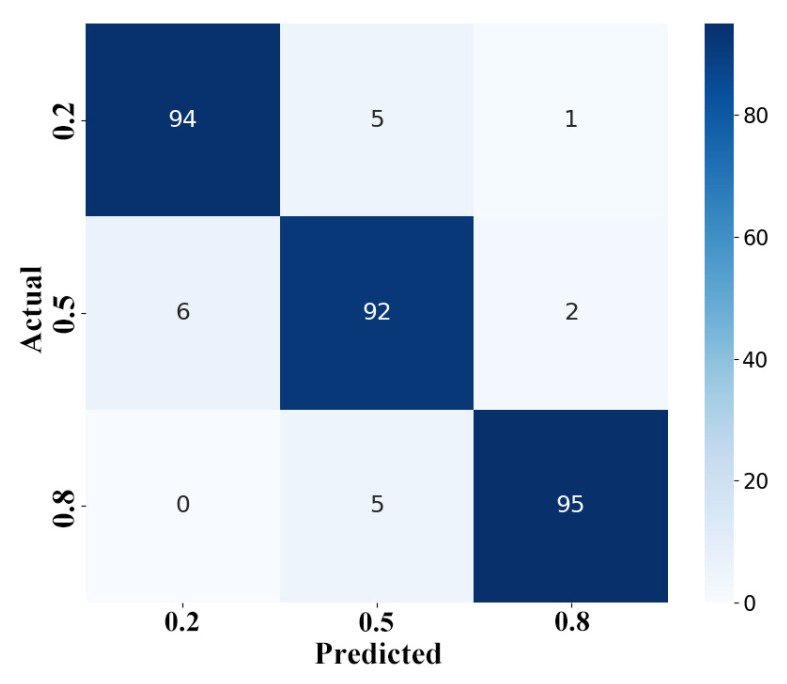
Confusion matrix using the SVM method.

After determining the optimal working frequency, we collected full-path S_21_ data at a PCC of 0.2, 0.5, and 0.8 kg/kg, respectively, and calculated their L1 distances. Using the SVM model, successful classification was achieved. The confusion matrix, presented in Figure 8, demonstrated that the majority of the predicted values were accurately classified, thereby validating the effectiveness of the prototype design and the methodology for the microwave measurement of PCC.

### 4.2. Field Experiments

The coal-fired unit typically utilizes a direct-firing pulverizing system. Raw coal is transported from the coal bunker to the coal feeder via a conveyor belt. Subsequently, the raw coal is fed into the coal mill in a controlled, continuous, and adjustable manner for pulverization. The pulverized coal is then conveyed to the boiler through the coal pipeline with the aid of heated primary air for combustion.

**Figure 9:**
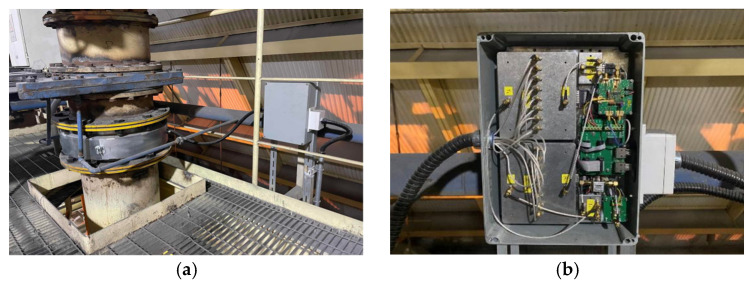
Installation outcomes. (**a**) Overall system overview; (**b**) hardware of the high-speed microwave signal routing module.

After the preliminary certification of the prototype experiments, we conducted tests directly in the coal-fired units at the power plant to evaluate the system’s performance under the field conditions. The multi-frequency microwave sensing system was installed on the coal pipelines of several coal mills. As shown in Figure 9, the outcomes of the installation are illustrated.

Currently, there is no consensus on the methods for measuring the concentration of gas–solid two-phase flows, which complicates the direct determination of absolute concentrations. Consequently, during the experiments, we monitored both the primary air flow rate of the coal mill and the coal quantity from the coal feeder, as recorded by the DCS system. The PCC at each moment was calculated using the following formula:

(10) $$PCC=\frac{M_{Coal}}{V_{air}},$$

(11) $$V_{air}=\frac{Q_{air}}{\rho_{air}},$$

where $M_{Coal}$ is the coal quantity from the coal feeder, $V_{air}$ is the primary air flow volume, and $Q_{air}$ is the primary air flow rate.

After integrating the system into the field environment, adjustments were made to the factors, such as sensor size and fabrication materials to better suit the conditions, as detailed in Table 4. The ability to identify an appropriate working frequency range for the scenario significantly influenced the measurement results.

A frequency sweep was conducted across the range of 0.55 to 3 GHz. The L1 distance metric was used to assess the signal attenuation at each frequency through the pulverized coal flow, with the results presented in Figure 7b. The results clearly indicate that the microwave signals experienced the greatest attenuation within the frequency range of 1.2 to 1.5 GHz. This suggests higher sensitivity to pulverized coal within this range, which provides a basis for frequency selection. Due to hardware constraints, only a single VCO could be used to generate the frequency. As a result, we selected the ROS-2500+, which offered a frequency sweeping from 1.2 GHz to 2.5 GHz with a voltage range of 0–11 V.

**Figure 10:**
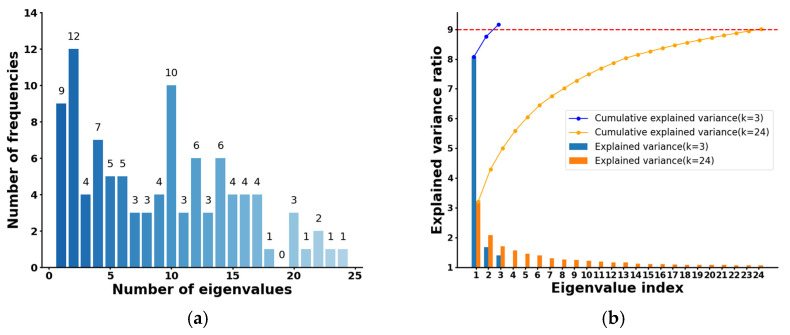
(**a**) Distribution of frequencies by number of eigenvalues; (**b**) cumulative explained variance chart for different frequencies.

To address the redundant information in the excess frequencies and the non-linear relationship between the S_21_ parameters and solid phase concentration, a frequency selection method based on principal component analysis (PCA) and orthogonal matching pursuit (OMP) [33] was employed. By varying the voltage from 0 to 10 V in increments of 0.1 V using a VCO during each acquisition, extensive frequency domain information was collected. Additionally, signal path switching was managed by a high-speed microwave signal routing module, resulting in a comprehensive 240-dimensional dataset across 101 frequencies, which formed the raw data matrix. Eigenvalue decomposition was performed for each frequency to derive multiple eigenvectors and their explained variances. We ensured that the eigenvectors for each frequency retained more than 80% of the information content from the raw data matrix, i.e., the cumulative explained variance exceeded 80%. As illustrated in Figure 10a, the number of eigenvectors k was generally below 24 for most of the frequencies, demonstrating that PCA effectively reduced the dimensionality of the high-dimensional full-path data. The cumulative explained variance was plotted for frequencies corresponding to eigenvector counts of k = 3 and k = 23, respectively. As shown in Figure 10b, when k was smaller, each eigenvector contained more information, allowing for the characterization of the original dataset with less data. This effectively reduced the computational requirements.

**Figure 11:**
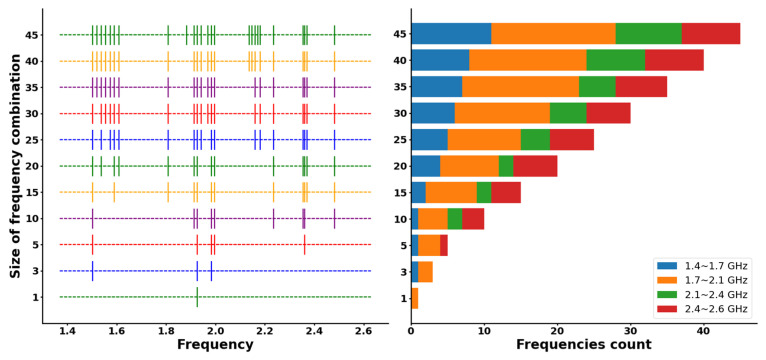
Eigenvector distribution for frequency combinations.

After applying PCA, 25 frequencies with k ≤ 3 were selected, resulting in a dataset comprising 45 sets of eigenvectors. Subsequently, OMP was used to select the optimal subset of these eigenvectors. The distribution of eigenvectors across various sizes of frequency combinations is illustrated in Figure 11. It is evident that these eigenvectors exhibit clustering in specific frequency bands, suggesting that the measurements in these bands more effectively represent the actual value.

**Figure 12:**
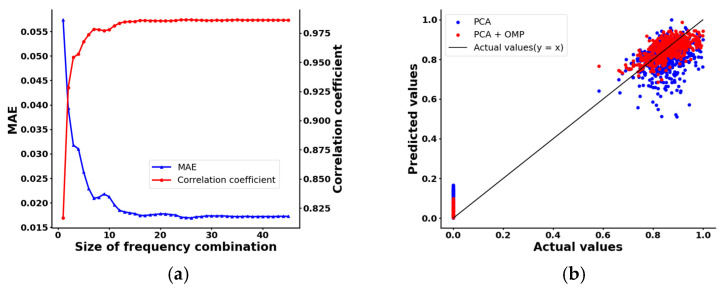
(**a**) Effect of frequency combination size on prediction result; (**b**) comparison of prediction results at different stages of frequency selection methods.

The mean absolute error (MAE) and correlation coefficient between the predicted and the actual values for various sizes of frequency combinations are shown in Figure 12a. As the size of the frequency combination increased, the MAE decreased while the correlation coefficient increased. This trend indicates that incorporating more eigenvectors improves the accuracy of the predictions. However, beyond a certain point, both the MAE and correlation coefficient tend to stabilize, suggesting that some eigenvectors become redundant and have minimal impact on the prediction results.

Based on the results of the OMP calculations, we could determine the optimal size N of the frequency combination S by identifying the point at which both the MAE and the correlation coefficient stabilized. Considering both the OMP results and the efficiency of data collection and processing, we concluded that N = 16. Figure 12b presents a preliminary comparison of prediction results from two stages of the frequency selection method, using PCA alone versus integrating PCA with OMP. After normalizing these predictions through min–max normalization, we visualized their distances from the actual values. The predictions from PCA combined with OMP were closer to the actual values. An error analysis revealed that the MAE for PCA alone can be as low as 7%, whereas the PCA+OMP method achieves a MAE of 3.8%. This demonstrates that combining PCA with OMP effectively integrates multi-frequency information, thereby improving prediction accuracy.

**Figure 13:**
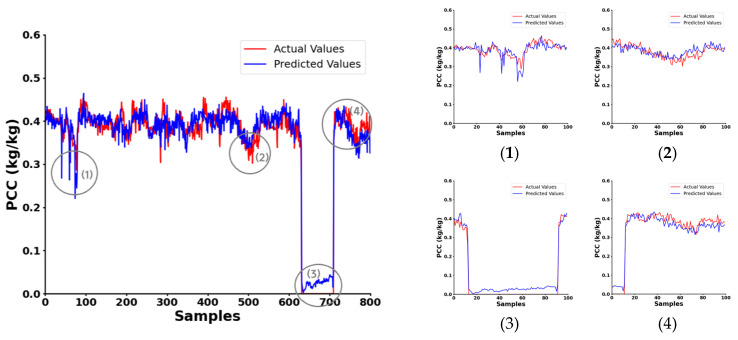
Test results on the C# coal mill.

We conducted on-site monitoring of the C# coal mill, as illustrated in Figure 13, which displays the predicted results from our frequency selection method alongside the actual PCC data. This comparison indicates that the system effectively captures the trend of PCC variations within the 0 to 0.5 kg/kg range. We have highlighted several notable regions with circles and included an enlarged view of these areas. In these marked regions, the slopes of the predicted values closely aligned with those of the actual measurements. Even when the conveying of pulverized coal ceased and the PCC approached zero, the multi-frequency microwave sensing system continued to detect changes promptly. An error analysis revealed a MAE of 2.1%, demonstrating the system’s effectiveness and reliability.

**Figure 14:**
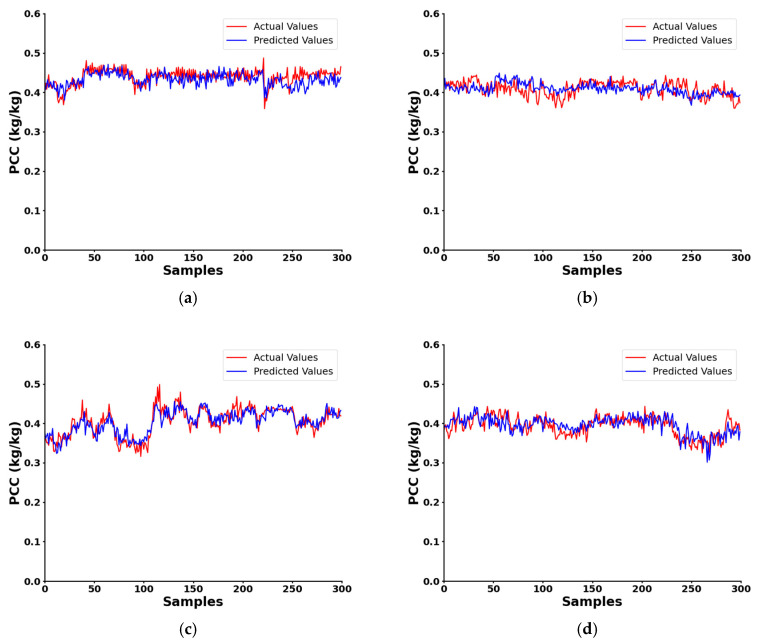
Test results on other coal mill: (**a**) A#; (**b**) B#; (**c**) D#; (**d**) E#.

Identical tests were conducted on various coal mills within the same unit to evaluate the reproducibility of the proposed method. The results, shown in Figure 14, indicate that the system effectively estimated PCC within the typical range of 0.3 to 0.5 kg/kg, which is commonly observed in power plants. A comparison of these estimates with the PCC data provided by the DCS system reveals that the predicted results closely align with the actual values in terms of trends. Furthermore, at points with more pronounced slopes, the predicted and actual values exhibited simultaneous peaks and troughs, further confirming the system’s accuracy.

A numerical analysis of the experimental results was conducted, focusing on error and correlation. As shown in Table 5, the MAE for nearly all the coal mills did not exceed 2.1%, with the best result reaching 1.41%. This indicates a high level of consistency between the predicted and actual values, thereby validating the repeatability and effectiveness of the multi-frequency microwave sensing system. Furthermore, it was observed that the amplitude of changes in the PCC was positively correlated with the correlation coefficient. This suggests that when the amplitude changes are substantial, measurement errors in the system are negligible, resulting in an increased correlation coefficient. Conversely, when the amplitude changes are minor, the relative error increases, and the effects of the amplitude changes on the results become less pronounced, leading to a decrease in the correlation coefficient. Thus, the system can achieve higher measurement accuracy under the conditions of significant PCC variation, thereby maintaining the accuracy and reliability of the predictions in dynamic environments.

## 5. Conclusions

In summary, this study demonstrates the effective application of a novel, non-contact, non-invasive sensing technology for PCC measurement that combines a frequency selection method with a microwave sensor. A hardware system was developed to flexibly control both the working frequency and signal path, enabling full-path insertion loss (S21) measurements across multiple frequencies, which served as inputs for data processing. A frequency selection method using PCA and OMP algorithms further filtered out redundant frequency signals, extracting key information from the high-dimensional PCC dataset.

This system offers notable advantages: its cross-sectional measurement capability effectively captures variations across the entire pulverized coal flow, addressing the inconsistencies in coal distribution that single-path measurement systems often encounter. Furthermore, its full-frequency measurement approach leverages frequency–domain information, achieving higher accuracy in PCC predictions than single-frequency methods. Additionally, its non-intrusive design ensures the system does not interfere with coal flow, making it well-suited for integration in industrial environments.

Experimental results show that the multi-frequency microwave sensing system effectively utilizes frequency information, employing a multi-frequency strategy that significantly improves prediction accuracy and demonstrates the feasibility of quantitative PCC measurement. If the original data changes significantly, the system may face parameter failure. Long-term measurements and recordings in coal-fired power plants indicate that the system reliably captures PCC variations within the 0 to 0.5 range, achieving an optimal MAE of 1.41%. However, the system is based on parameters derived from a predefined dataset, which limits its adaptability. Future work could focus on reducing the system’s error rate and enhancing its adaptive capabilities, allowing it to adjust dynamically to environmental changes such as temperature fluctuations or coal accumulation.

## Figures and Tables

**Table 1:** Performance comparison with multi-frequency systems in the literature.

Ref.	Method	Frequency Range (GHz)	Selection Method	Pros	Cons	Application
[17]	Resonant Cavity Methods	1.5~1.8	Sweep range of Vector Network Analyzer (VNA)	- Strong interpretability - Low environmental impact	- Calibration required - Narrow bandwidth	Industrial: gas–solid two-phase flow
[23]	MWT Methods	3~6	Compare single antenna vs. co-located measurements; choose frequency with minimal coupling difference	- Strong interpretability - Simple Calibration	- Additional measurements required - High hardware requirements	Biomedical: dielectric phantoms
[24]	Transmission Methods	2~10	Random Forest-Recursive Feature Elimination (RF-RFE) + Majority Voting Method (MVM)	- Data-driven	- Lack of interpretability - High data dependency	Agriculture: corn moisture
This Work	Transmission Methods	1.5~2.5	Principal Component Analysis (PCA) + Orthogonal Matching Pursuit (OMP)	- Data-driven - Effective for high-dimensional data	- High data dependency - Narrow bandwidth - Limited adaptability to environmental changes	Industrial: Pulverized Coal Concentration (PCC)

**Table 2:** Parameters of the chips used in the switch matrix.

Device Type	Part #	Manufacturer	City and Country	Switching Time (ns)
SPDT RF Switch	M3SWA2-63DRC+	Mini-Circuits	Brooklyn, NY, USA	6
3 TO 8 Line Decoder Demultiplexer	74HC138	Diodes	Plano, TX, USA	12

**Table 3:** Correlation coefficient for different experimental scenarios.

Experiments Scenario	Correlation Coefficient					
	Test 1	Test 2	Test 3	Test 4	Test 5	Test 6
Prototype Experiments	1	0.892	0.911	0.874	0.840	0.878
Field Experiments	1	0.923	0.950	0.952	0.987	0.927

**Table 4:** Sensor specifications for different experimental scenarios.

Experiments Scenario	Sensor Diameter (mm)	Outer Wall Material	Substrate Layer Material
Prototype Experiments	150	Aluminum	Epoxy Resin
Field Experiments	508	Carbon Steel	PTFE

**Table 5:** Numerical analysis of test results on different coal mills.

	A#	B#	C#	D#	E#
Mean absolute error (%)	1.46	1.58	2.07	1.41	1.55
Correlation coefficient	0.57	0.31	0.98	0.85	0.66

## Data Availability

The original contributions presented in this study are included in this article; further inquiries can be directed to the corresponding authors.
